# mTOR Mysteries: Nuances and Questions About the Mechanistic Target of Rapamycin in Neurodegeneration

**DOI:** 10.3389/fnins.2020.00775

**Published:** 2020-07-29

**Authors:** Nicholas G. Norwitz, Henry Querfurth

**Affiliations:** ^1^Department of Physiology, Anatomy and Genetics, University of Oxford, Oxford, United Kingdom; ^2^Department of Neurology, Tufts Medical Center, Boston, MA, United States

**Keywords:** Alzheimer’s disease, antagonistic pleiotropy, autophagy, insulin/Akt, mTOR, Parkinson’s disease

## Abstract

The mechanistic target of rapamycin protein complex, mTORC1, has received attention in recent years for its role in aging and neurodegenerative diseases, such as Alzheimer’s disease. Numerous excellent reviews have been written on the pathways and drug targeting of this keystone regulator of metabolism. However, none have specifically highlighted several important nuances of mTOR regulation as relates to neurodegeneration. Herein, we focus on six such nuances/open questions: (1) “Antagonistic pleiotropy” – Should we weigh the beneficial anabolic functions of mTORC1 against its harmful inhibition of autophagy? (2) “Early/late-stage specificity” – Does the relative importance of these neuroprotective/neurotoxic actions change as a disease progresses? (3) “Regional specificity” – Does mTOR signaling respond differently to the same interventions in different brain regions? (4) “Disease specificity” – Could the same intervention to inhibit mTORC1 help in one disease and cause harm in another disease? (5) “Personalized therapy” – Might genetically-informed personalized therapies that inhibit particular nodes in the mTORC1 regulatory network be more effective than generalized therapies? (6) “Lifestyle interventions” – Could specific diets, micronutrients, or exercise alter mTORC1 signaling to prevent or improve the progression neurodegenerative diseases? This manuscript is devoted to discussing recent research findings that offer insights into these gaps in the literature, with the aim of inspiring further inquiry.

## Introduction

Neurodegenerative diseases are an accelerating pandemic. The burden of Alzheimer disease (AD) alone is staggering and climbing at a precipitous rate. 5.8 million Americans over the age of 65 suffer from AD, a number that is expected to triple to 13.8 million by 2050 (Alzheimer’s Association, 2020). AD is not alone in its ascent. Parkinson’s disease (PD), the second most common form of neurodegeneration, is increasing in prevalence at a similarly alarming rate (Rocca, 2018). As there are currently no effective long-term treatments for these diseases, new therapies are desperately needed. One potential molecular target of such therapies is the mechanistic target of rapamycin complex 1 (mTORC1), a nutrient sensor and metabolic regulator heavily implicated in the process of aging (Sharp and Strong, 2010; Papadopoli et al., 2019; Heras-Sandoval et al., 2020).

**FIGURE 1:**
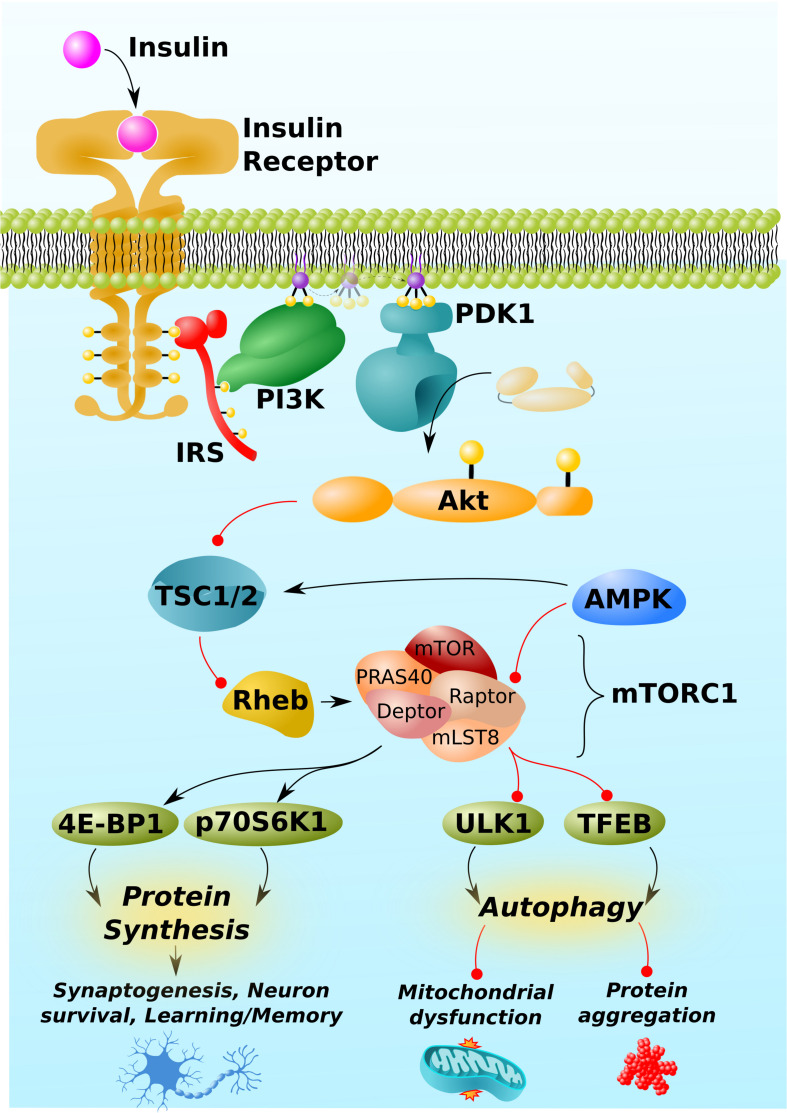
mTORC1 pathway and regulation. mTORC1 is activated by insulin. Insulin/Akt signaling inhibits TSC1/2, thereby permitting the activation of the GTP-binding protein, Rheb. Rheb is the proximal activator of mTORC1. AMPK inhibits mTORC1 activity through indirect and direct mechanisms, phosphorylating TSC1/2 and the Raptor regulatory component of mTORC1. (Other trophic factors and pathways beyond insulin/Akt and AMPK, not shown for simplicity, also regulate mTORC1). mTORC1 downstream targets include proteins involved the mRNA translation, 4E-BP1 and p70S6K1, and those involved in autophagy, such as the initiator of autophagy, ULK1, and the master regulator of lysosomal biogenesis, TFEB. By regulating the activity of these and other proteins, mTORC1 promotes protein synthesis, which is required for synaptogenesis, learning, and memory, but can also impair autophagy, leading to mitochondrial dysfunction and neurotoxic protein aggregation (Aβ, phospho-tau, α-synuclein, etc.). Black arrows and red lines respectively represent positive and negative regulation.

While this manuscript will be primarily devoted to discussing and gaps in the literature surrounding mTORC1, a succinct overview of mTOR signaling and regulation is warranted as a preface to this discussion and is depicted in Figure 1 [For a more comprehensive overview, Heras-Sandoval et al. (2020) recently published an excellent review on mTOR signaling, regulation, and drug-targeting]. mTORC1 is composed of the proteins mTOR kinase and its regulator protein, Raptor, as well as mLST8, PRAS40, and Deptor. Its primary function is to sense intracellular nutrient status and extracellular trophic factors [including, but not exclusive to insulin, shown in Figure 1 as an example], integrate these signals, and ultimately regulate the balance between cells’ anabolic and catabolic processes. Specifically, mTORC1 is a positive regulator of protein synthesis and negative regulator of autophagy.

mTORC1 itself is regulated positively by insulin-signaling and negatively by AMPK. Insulin/Akt signaling inhibits the protein complex, TSC1/2, which itself prevents the conversion of the mTORC1 activator, Rheb, into its active GTP-bound form (Inoki et al., 2002; Hers et al., 2011). Insulin/Akt signaling turns off TSC1/2, thereby activating Rheb and mTORC1. By contrast, AMP-activated protein kinase (AMPK) activates TSC1/2 (Inoki et al., 2003) and directly inhibits mTORC1 by phosphorylating Raptor (Gwinn et al., 2008). In brief, the respective growth and preservation functions of insulin and AMPK align with their respective stimulatory and inhibitory regulations of mTORC1.

mTORC1 promotes protein synthesis by phosphorylating and activating the downstream targets, 4E-BP1 and p70S6K1, which directly promote the initiation and elongation phases of translation (Graber et al., 2013). Critically, mTORC1-mediated anabolic signaling promotes the development of neuronal synapses (Dwyer and Duman, 2013, in part, by responding to established neuronal growth factors like BDNF; Takei et al., 2004) and inhibits apoptosis (Chen et al., 2010; Chong et al., 2013). Through these two mechanisms, mTORC1 activity has the potential to promote learning and memory and protect against neurodegeneration. Correspondingly, excessive inhibition of mTORC1 can impair learning and memory and permit neuronal death (Blundell et al., 2008; Belelovsky et al., 2009; Gafford et al., 2011; Jobim et al., 2012; Graber et al., 2013).

Despite these potentially positive functions of mTORC1 signaling in the brain, far more attention has been paid to its negative regulation of autophagy, an intracellular recycling process essential to maintaining neuronal integrity and protecting against neurodegenerative diseases (Oddo, 2012; Sarkar, 2013; Heras-Sandoval et al., 2020). mTORC1 inhibits autophagy at multiple levels, including the inhibitory phosphorylation of ULK1 and transcription factor EB (TFEB), which respectively initiate autophagy and promote the lysosomal biogenesis required to break down the contents of autophagosomes (Kim et al., 2011; Napolitano et al., 2018).

Importantly, multiple independent human post-mortem studies confirm levels of phosphorylated mTOR and its downstream targets are elevated in the AD brain as compared to those of controls (An et al., 2003; Li et al., 2004, 2005; Griffin et al., 2005). Dysregulated autophagy is also a hallmark of multiple neurodegenerative conditions (Fujikake et al., 2018), which is not surprising because autophagy is required to prevent the accumulation of toxic intracellular protein aggregates that contribute to neurodegenerative diseases, such as amyloid-β (Aβ) (Nixon, 2007; Nilsson et al., 2013; Yang et al., 2014), phospho-tau (Hamano et al., 2008; Kruger et al., 2012; Wang and Mandelkow, 2012), α-synuclein (Webb et al., 2003; Lee et al., 2004; Xilouri et al., 2016), and mutant huntingtin (Martin et al., 2015). Autophagy is also required to recycle mitochondria and prevent mitochondrial dysfunction (Chakravorty et al., 2019; Li et al., 2019a), another hallmark of neurodegenerative diseases, and one which can further lead to the pathologies of oxidative stress and inflammation (Lopez-Armada et al., 2013; Norwitz et al., 2019a, b). Given these data and the clinical burden of neurodegenerative disease, it’s reasonable that translational research generally focuses on the inhibition of mTOR (and promotion of autophagy), rather than on its activation.

## Antagonistic Pleiotropy

“Antagonistic pleiotropy” is a term typically used to refer to an evolutionary tradeoff between fitness in an organism’s early life at the expense of health later in life (Schmeisser and Parker, 2019). An example of antagonistic pleiotropy is the *ApoE4* allele, the leading genetic risk factor of AD (Yamazaki et al., 2019). This allele sensitizes the immune system and protected ancestral humans from infections that compromised reproductive fitness and cognition (Vasunilashorn et al., 2011; Trumble et al., 2017). Further relevant to modern contexts, *ApoE4* is associated with accelerated neurodevelopment (Wright et al., 2003) and improved memory during youth (Mondadori et al., 2007).

Another example of possible antagonistic pleiotropy in neurodegenerative disease is that of adiponectin (APN), a hormone secreted by adipose tissue. APN has broad beneficial functions on metabolism, including stimulating neurogenesis, and is generally thought to be neuroprotective (Zhang et al., 2011, 2016). On the other hand, APN can induce astrocyte mediated neuroinflammation (Wan et al., 2014), oxidative stress (Fujimoto et al., 2010), and plasma levels of APN are correlated with the severity of cognitive decline and Aβ accumulation (Wennberg et al., 2016). [Waragai et al. (2020) provide a comprehensive overview of antagonistic pleiotropy with regards to APN].

Hashimoto et al. (2018) have even proposed that amyloidogenic proteins, including Aβ in AD and α-synuclein in PD, might exhibit antagonistic pleiotropy. They hypothesize that the heterogeneity of amyloidogenic aggregates reflects the heterogeneity of metabolic stressors to which the human brain is exposed, and that specific amyloidogenic aggregates may serve to “precondition” the brain against future toxic exposures (Hashimoto et al., 2018). In effect, Aβ and α-synuclein could serve, in youth, as adaptive hormetic stressors. [As an aside, the Aβ/α-synuclein antagonistic pleiotropy hypothesis is intertwined with the “evolvability hypothesis” of amyloidogenic proteins, which is beyond the scope of this piece and reviewed by Hashimoto et al. (2018)].

The moral of these examples – *ApoE4*, APN, and Aβ/α-synuclein – is that the trade of better health and cognition during youth, at the expense of cognition during non-reproductive years, was evolutionarily judicious. Furthermore, each these examples would not have been specifically mentioned if they did not plausibly involve mTORC1. With respect to *ApoE4*, mTORC1 activates pathways that promote synaptogenesis and neuronal development, which would benefit cognition during youth at the expense of decreased autophagy and increased risk of accumulating mitochondrial damage and neurotoxic protein aggregates over time, as in the case of *ApoE4* (Wright et al., 2003; Mondadori et al., 2007). Indeed, the *ApoE4* genotype is associated with elevated mTOR signaling (Li et al., 2019b). APN has been shown to induce oxidative stress in an mTORC1-dependent manner by modulating both insulin and AMPK signaling (Fujimoto et al., 2010; Figure 1). And, of course, mTORC1 activity is assumed to be culpable for dysfunctional autophagy and accumulation of neurotoxic protein aggregates in neurodegenerative diseases, as noted in the introduction. Thus, mTORC1 may be a keystone player of antagonistic pleiotropy in neurodegenerative disease.

Consideration of antagonistic pleiotropy is important for evaluating the preventative value of inhibiting mTORC1 prior to the development of symptoms. No doubt, it’s important to prevent the development of the pathologies underlying neurodegenerative diseases, which are established decades before symptoms develop (Braak et al., 2003; Dickson et al., 2010; Hoglund et al., 2017). But when and by how much? During mid-life, should one strive for mTORC1 inhibition, or value activating mTORC1 in a cyclic manner in order to build neural networks and increase her/his cognitive reserve, thus protecting against cognitive decline later in life? These are open questions.

## Early/Late-Stage Specificity

Although inhibiting mTORC1 to increase autophagy (and therefore clear damaged mitochondria and protein aggregates) may seem like a prudent intervention for neurodegenerative diseases, that may not be universally true. What if a disease progresses past a threshold beyond which the pathology is too well-established to be meaningfully improved by an upregulation in autophagy? For instance, the mTORC1 inhibitor, rapamycin, does not reverse pathology or benefit cognition in late-stage AD models (Majumder et al., 2011). More importantly, because mTORC1 can inhibit apoptosis by activating p70S6K, which itself inhibits the pro-apoptotic protein BAD (Harada et al., 2001; Castedo et al., 2002), what if inhibiting mTORC1 beyond this hypothetical threshold increases apoptotic cell death?

Evidence consistent with this hypothesis is provided by multiple independent cell and rodent models of PD. These models of established late-stage disease suggest that increasing, rather that decreasing, mTOR activity could be beneficial under certain circumstances. In MPP^+^-treated SH-SY5Y cells, activation of mTOR with cannabidiol led to protection against MPP^+^-induced cell death (Gugliandolo et al., 2020). In genetic and pharmacologic mouse models, upregulation of mTOR signaling (through PTEN ablation) is likewise associated with less cell death and improved symptomology (Domanskyi et al., 2011). A limitation of these early PD studies is that they do not involve α-synuclein accumulation, which may better recapitulate the human form of the disease and relative importance of autophagy therein. Nevertheless, given the knowledge that mTORC1 can inhibit apoptosis, and distinct possibility that there may be a point past which activation of autophagy is insufficient to improve disease course (Majumder et al., 2011), it’s worth questioning whether mTORC1 inhibition could actually be harmful in late-stage neurodegenerative disease.

## Regional Specificity

In addition to considering the temporal dimension (early/late-stage disease), it’s important to consider the spatial dimension. As the brain is partitioned into networks, nuclei, and cell types, a given intervention may impact one region differently than another. For example, Ramalingam et al. (2019) discovered that rotenone injections (used to generate murine models of PD) oppositely impact mTORC1 activity in different regions of mouse brains, increasing activity in the midbrain and decreasing activity in the striatum. Lifestyle interventions (more on this below), such as exercise, may also alter mTOR activity in a region-specific manner. In mice, wheel running regulates mTORC1 signaling most strongly in the nucleus accumbens and hippocampus, as compared to other brain regions (Lloyd et al., 2017). This is notable because atrophy of nucleus accumbens and hippocampus is most strongly associated with AD (Nie et al., 2017).

The data are nascent but sufficient to issue caution. What if a PD patient suffering from substantia nigra atrophy were treated with an mTORC1 inhibitor based on a rationale from data collected from hippocampal pathology? What if a frontotemporal dementia (FTD) patient suffering from primarily temporal lobe pathologies was treated with an mTORC1-targeting drug based on frontal lobe data? As there is limited evidence to support that mTORC1 responds consistently to a wide range of interventions across brain regions, and some evidence to the contrary, it’s responsible to not overgeneralize and assume globalized impact on the brain. More research needs to be conducted on the region-specific impacts of different mTORC1-directed interventions.

## Disease Specificity

While many neurodegenerative diseases share several key core pathologies, including mitochondrial dysfunction, protein aggregation, oxidative stress, and inflammation, it’s also important to consider disease-specific aspects of neurometabolim that could interact with mTORC1. For example, Zhuang et al. (2020) recently discovered that TFEB activity (which stimulates lysosomal biogenesis and promotes autophagy) is increased in a 6-OHDA-treated SH-5YSY model of PD, as well as in dopaminergic neurons, and that TFEB activity is calcium/calcineurin-dependent. This is important because PD is characterized by loss of substantia nigra pars compacta neurons, which exhibit a unique form of calcium pacemaking activity not seen in most other neurons. This suggests that regulation of autophagy may be different in the brain region most affected by PD as compared to brain regions impacted in other diseases.

Another example is Amyotrophic Lateral Sclerosis (ALS), which can be caused by loss-of-function mutations in the *UBQLN2/4* genes. While the products of these genes, ubiquilin proteins, are known best as components of the ubiquitin-proteasome system, they are also important in autophagy. Specifically, ubiquilins are required to maintain the vacuolar H^+^-ATPase function that acidifies lysosomes (Senturk et al., 2019). In a scenario in which mTORC1 were inhibited to induce autophagy in ALS, induction of autophagy and lysosomal biogenesis may be increased (Figure 1), but if lysosomes are not sufficiently acidic to destroy the contents of the autophagosome, the contents could accumulate and exacerbate cellular stress. Therefore, inhibiting mTORC1 to upregulate autophagy could impair autophagic flux, leading to a back-up of components, and be harmful in such genetic cases of ALS.

## Personalized Therapy

There is a need for informed, disease-specific interventions. In this section, we provide three hypothetical examples of personalized interventions involving mTORC1. These will include glutamatergic antagonism for Huntington’s disease (HD) (Abd-Elrahman and Ferguson, 2019), metformin treatment for multiple sclerosis (MS) (Sanadgol et al., 2019), and SMCR8-centered therapy for ALS and FTD (Lan et al., 2019).

Glutamate hyperactivity plays a prominent role in HD (Andre et al., 2010) and can activate mTORC1 via the mGluR5-PDK1-Akt-mTORC1 pathway (Abd-Elrahman and Ferguson, 2019). Correspondingly, Abd-Elrahman and Ferguson (2019) recently demonstrated, in a mouse model of HD, that antagonism of the mGluR5 metabotropic glutamate receptor can correct overactive mTORC1 signaling and, consequently, increase autophagic clearance of mutant huntingtin protein. The authors of this paper also point out that huntingtin aggregates sequester the transcription factor, CREB, leading to a down regulation of neuroprotective BDNF. They show that mGluR5 inhibitors not only clear pathological aggregates, but also increase BDNF expression (Abd-Elrahman and Ferguson, 2019). Therefore, mGluR5 antagonism, by inhibiting hyperactive mTORC1, could simultaneously promote the clearance of pathological huntingtin aggregates and increase neurotrophic factor signaling.

MS is characterized by demyelination of nerve cell axons. As oligodendrocytes are responsible for building myelin sheaths within the central nervous system, a goal of MS treatments is to boost oligodendrocyte renewal and remyelination. In a cuprizone-challenge mouse model of MS, Sanadgol et al. (2019) recently reported that the diabetes drug, metformin, did precisely that: it increased oligodendrocyte renewal and remyelination. These beneficial effects were mediated by a direct stimulatory interaction between metformin and AMPK, and subsequent inhibition of mTORC1 (Sanadgol et al., 2019; Figure 1). Thus, metformin is a candidate for an mTORC1-targeting therapy for MS.

Mutations in the *C9orf72* gene are the leading cause of inherited ALS and FTD. Only recently was it discovered that another protein, SMCR8, complexes with the C9orf72 protein to form a heterodimer that negatively regulates mTORC1 activity (Lan et al., 2019). Furthermore, a *SMCR8*-deficient mouse model recapitulates the *C9orf72*-deficient phenotype, leads to a decrease in C9orf72 protein, and is associated with upregulation of mTORC1 activity and decreased autophagy (Lan et al., 2019). Future treatments for genetic causes of ALS and FTD might consider SMCR8 therapy or other interventions that target the SMCR8-mTORC1-autophagy axis.

These examples were chosen because HD, MS, ALS, and FTD are lesser studied than AD and PD. However, the same personalization principle applies to all conditions in which mTORC1 plays a role. In PD, for example, levodopa-induced dyskinesia is thought to be induced by D1-receptor-mediated phosphorylation of mTORC1, a hypothesis supported by the fact that genetic variability in mTOR pathway components is associated with PD dyskinesia (Zhu et al., 2019). The development of useful future interventions for neurodegenerative disorders would benefit from a deeper consideration of the interactions between mTORC1 signaling and disease/patient-specific mechanisms.

## Lifestyle Interventions

Two reasons most neurodegenerative diseases are refractory to treatment are that interventions may be initiated too late in the disease process and/or are too specific. These limitations are a function of the pharmacologic approach to neurodegenerative disease in which symptomatic patients, who have usually been afflicted by the underlying disease for years to decades, are prescribed drugs not available for prevention during the preclinical stage. Certainly, drugs have their place. But to quell the neurodegenerative disease pandemic will require universally accessible preventative measures based on safe lifestyle interventions, including diet and exercise. Evidence suggests such interventions could operate, in part, through mTORC1-mediated mechanisms.

Turmeric is the best-studied nutraceutical for neurodegenerative diseases. In a genetic mouse model of AD, turmeric’s active component, curcumin, inhibited mTORC1 to increase autophagy and prevent Aβ accumulation (Wang et al., 2014a). Correspondingly, curcumin-induced inhibition of mTORC1 protected against memory impairments in this model (Wang et al., 2014a). A more specific dietary example would be the mineral manganese in HD. As manganese deficiency might contribute to the pathogenesis of HD by affecting the insulin/Akt/mTORC1 pathway, correcting a simple micronutrient deficiency could be protective in some cases of HD (Bryan and Bowman, 2017). A third example is that of PPARs, a family of transcription factors that can inhibit mTORC1 and promote autophagy to protect against neurodegenerative disease (San et al., 2015; Heras-Sandoval et al., 2020). Many nutrients and their derivates activate PPARs, including oleoylethanolamide derived from oleic acid in olive oil (Rodriguez de Fonseca et al., 2001; Fu et al., 2005) and the monoterpenes carvacrol and thymol found in mint family plants (basil, mint, rosemary, sage) (Hotta et al., 2010; Rigano et al., 2017). Curcumin, manganese, and dietary PPAR activators are just three examples of nutraceuticals from different classes that, when combined in a well-formulated diet and with other dietary mTOR regulators (Wang et al., 2014b; Rigano et al., 2017), could have a meaningful impact on cognitive longevity.

In addition to nutraceuticals and micronutrients, shifts in macronutrient intake can also impact mTORC1 activity. The most evident examples are intermittent fasting and high-fat, low-carbohydrate ketogenic diets, which can modulate mTORC1 activity through at least three mechanisms. First, fasting and ketogenic diets diminish insulin-mediated mTORC1 activation. Second, they activate AMPK (by altering the AMP/ATP ratio and causing glycogen depletion) to inhibit mTORC1 and induce autophagy (Alirezaei et al., 2010; Miller et al., 2018). Third, fasting and ketogenic diets share the common feature of stimulating hepatic production of the ketone body, β-hydroxybutyrate, which itself is a signaling molecule that regulates mTORC1 (Li et al., 2017; Newman and Verdin, 2017; Norwitz et al., 2019a). Interestingly, it has recently been demonstrated that both short-term ketogenic diets and acute administration of exogenous β-hydroxybutyrate improve a marker of brain aging called “brain network stability,” in contrast to standard Western diets and sugar which decrease network stability (Mujica-Parodi et al., 2020). Long-term prospective studies will need to be conducted to determine whether fasting and ketogenic diets are truly neuroprotective in humans. Nevertheless, these mechanisms and data coincide with the growing popularity of intermittent fasting and ketogenic diets as prevention or treatment strategies for neurodegenerative conditions (Roberts et al., 2017; Zhang et al., 2017; Shin et al., 2018; Sohn, 2018; Taylor et al., 2018, 2019; Broom et al., 2019; Norwitz et al., 2019a; Wlodarek, 2019; Mujica-Parodi et al., 2020; Soto-Mota et al., 2020).

Exercise is another lifestyle intervention that benefits brain health. Prospective cohort and randomized controlled studies have found that exercise reduces the risk of developing dementia by as much as 38% (Larson et al., 2006) and improves cognitive function in those already living with AD (Groot et al., 2016; Jia et al., 2019). Kou et al. (2019) recently published a compelling review arguing that the benefits of exercise on cognitive function and AD may be mediated by mTORC1 regulation. Even a cursory consideration of this hypothesis suggests it has merit. Exercise alters nutrient flux, trophic factor signaling, and can activate AMPK. Exercise can also correct overactive mTORC1 signaling to increase autophagy by correcting dysfunctional microRNA expression in a mouse model of AD (Kou et al., 2017; Chen et al., 2019). These particular studies focus on microRNA-34a, but there is reason to believe that exercise can influence mTORC1, autophagy, and cognitive aging by regulating a wide network of microRNAs (Kou et al., 2019). In another rodent model of AD, treadmill exercise decreased phospho-mTOR levels [Ser-2,448, Akt target residue (Nave et al., 1999)], increased autophagy, and completely rescued cognitive function on the Morris water maze test (Kang and Cho, 2015).

Dietary micronutrients, fasting and ketogenic diets, and exercise are but a few illustrative examples of lifestyle interventions that may interact with mTORC1 to modulate the course of neurodegenerative diseases. Additional therapies include probiotics to modulate the gut-brain axis, which has been heavily implicated in the development of neurodegenerative diseases (Sampson et al., 2016; Sochocka et al., 2019), and heat therapy to induce chaperone heat shock proteins [whose expression is at least partially mediated by mTORC1 (Sun et al., 2011)] that could promote the proper folding of amyloidogenic proteins (Singh et al., 2006, 2010; Laukkanen et al., 2017). At the present time, clinical studies examining the impact of lifestyle interventions on mTORC1 signaling for cognitive decline are few (Halikas and Gibas, 2018; Kou et al., 2019) and more research needs to be conducted in this area to inform holistic and universally available best practices for the treatment and prevention of neurodegenerative disease.

## Conclusion

**FIGURE 2:**
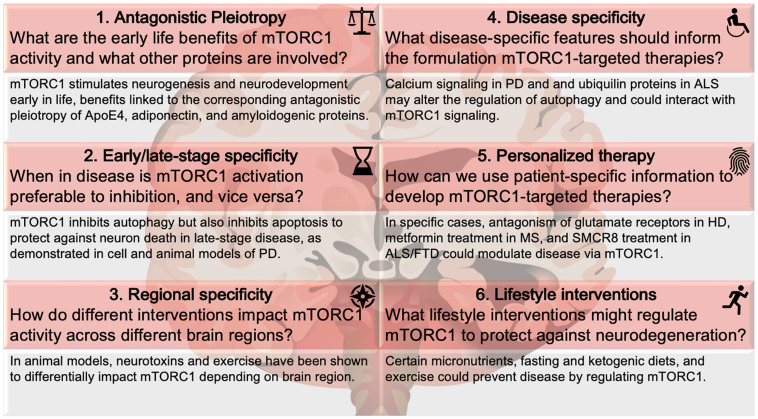
mTORC1 mysteries. Six nuances regarding mTORC1 in neurodegenerative disease. The questions and examples below each topic are illustrative, not comprehensive, of the literature covered in this review. Disease abbreviations: ALS, Amyotrophic Lateral Sclerosis; FTD, frontotemporal dementia; HD, Huntington’s disease; MS, multiple sclerosis; PD, Parkinson’s disease.

While references to the most pressing open questions are scattered throughout the abundant literature on mTOR and neurodegenerative disease, herein, we have consolidated these gaps in the literature (Figure 2). How do we balance the beneficial effects of mTORC1 against its negative effects? How does this balance shift with disease progression or brain region? How can we use knowledge of biochemical pathways, specific to diseases and even individual cases, to inform personalized therapy? And what universally available lifestyle interventions might help in the prevention of neurodegeneration? Consideration of these mTOR mysteries will inform future research.

## Author Contributions

Both authors contributed to this work.

## Conflict of Interest

The authors declare that the research was conducted in the absence of any commercial or financial relationships that could be construed as a potential conflict of interest.

## Abbreviations

A β: amyloid- β
AD: Alzheimer’s disease
Akt: protein kinase B
ALS: Amyotrophic Lateral Sclerosis
AMPK: AMP-activated protein kinase
APN: adiponectin
BAD: Bcl2 associated agonist of cell death
CREB: cAMP response element-binding protein
*C9orf72*: chromosome 9 open reading frame 72
Deptor: domain-containing mTOR-interacting protein
FTD: frontotemporal dementia
HD: Huntington’s disease
IRS: insulin receptor substrate
mGluR5: metabotropic glutamate receptor type 5
mLST8: mammalian lethal with SEC13 protein 8
MPP+: 1-methyl-4-phenylpyridinium
MS: multiple sclerosis
mTOR: mechanistic target of rapamycin
PD: Parkinson’s disease
PDK-1: phosphoinositide-dependent kinase-1
PI3K: phosphoinositide 3-kinase
PPAR: peroxisome proliferator-activated receptor
PRAS40: proline-rich Akt substrate of 40 kDa
PTEN: phosphatase and tensin homolog
p70S6K1: p70 ribosomal S6 protein kinase 1
Raptor: regulatory-associated protein of mTOR
Rheb: Ras homolog enriched in brain protein
SMCR8: Smith-Magenis syndrome chromosome region 8
TFEB: transcription factor EB
TSC1/2: Tuberous sclerosis protein-complex
*UBQLN2/4*: ubiquilin genes
ULK1: Unc-51-like kinase 1
4E-BP1: 4E-binding protein-1
6-OHDA: 6-hydroxydopamine.
